# The Investigation of Unexpected Arsenic Compounds Observed in Routine Biological Monitoring Urinary Speciation Analysis

**DOI:** 10.3390/toxics5020012

**Published:** 2017-05-20

**Authors:** Elizabeth Leese, Malcolm Clench, Jackie Morton, Philip H.E. Gardiner, Vikki A. Carolan

**Affiliations:** 1 Health and Safety Executive, Harpur Hill, Buxton, Derbyshire SK17 9JN, UK; jackie.morton@hsl.gsi.gov.uk; 2 Biomolecular Sciences Research Centre, Sheffield Hallam University, Howard Street, Sheffield S1 1WB, UK; m.r.clench@shu.ac.uk (M.C.); p.h.gardiner@shu.ac.uk (P.H.E.G.); v.a.carolan@shu.ac.uk (V.A.C.)

**Keywords:** arsenic, speciation, thioarsenical, thio-DMA, urine, exposure, ICP-MS, oxoarsenicals

## Abstract

This study investigates the identity of two unexpected arsenic species found separately in a number of urine samples sent to the Health and Safety Executive’s Health and Safety Laboratory for arsenic speciation (arsenobetaine, AB; arsenite, As^3+^; arsenate, As^5+^; monomethylarsonic acid, MMA^5+^; and dimethylarsinic acid, DMA^5+^). Micro liquid chromatography coupled to inductively coupled plasma mass spectrometry (µLC-ICP-MS) and electrospray time of flight tandem mass spectrometry (ESI-QqTOF-MS/MS) were used to identify the two arsenic peaks by comparison to several characterized arsenicals: arsenocholine, AC; trimethyl arsine oxide, TMAO; dimethylarsenoacetate, DMAA; dimethylarsenoethanol, DMAE; thio-dimethylarsinate, thio-DMA; thio-dimethylarsenoacetate, thio-DMAA and thio-dimethylarsenoethanol, thio-DMAE. The results from both the ICP-MS and ESI-QqTOF-MS/MS investigations indicate that the unexpected arsenic species termed peak 1 was thio-DMA. While the unexpected arsenic species termed peak 2 has yet to be identified, this investigation shows that it was not AC, TMAO, DMAA, DMAE, thio-DMA, thio-DMAA or thio-DMAE. This study demonstrates the incidence of unexpected arsenic species in both routine and non-routine urine samples from both workers and hospital patients.

## 1. Introduction

Identifying arsenic species in urine samples presents both an analytical and biological challenge, as the metabolism and biotransformation of arsenic compounds in humans is complex [1]. In part this is due to the fact that there is no exact agreed process for this human metabolic pathway of arsenic in the literature, with Hayakawa [2], Naranmandura [3] and Wang [4] all proposing slightly different biomethylation pathways. In 1945, the Challenger pathway [5] was first proposed in humans suggesting an initial reduction of arsenate to arsenite followed by steps of oxidation methylation and reduction methylation as follows: arsenate > arsenite > monomethylarsonic acid > monomethylarsonous acid > dimethylarsinic acid > dimethylarsinous acid (As^5+^ > As^3+^ > MMA^5+^ > MMA^3+^ > DMA^5+^ > DMA^3+^). More recent studies [2,3,4] have suggested that the pentavalent methylated species are not intermediates as presumed in the Challenger pathway but rather an end product of arsenic biotransformation. It has also been shown that the presence of the mammalian methyltransferase enzyme As3MT [6,7] (previously named Cyt19) [8] is crucial for arsenic metabolism, as it enables the transfer of the methyl donor S-adenosyl-L-methionine (SAM) in the presence of glutathione (GSH) [2,9]. It is suggested that the primary site of arsenic methylation in humans is the liver [8,10], because the liver retains and accumulates arsenic [11] in addition to being a rich source of GSH [12].

In the general population, the primary source of arsenic exposure is through ingestion from dietary and water sources. It is also possible that workplace exposure could occur in industries such as semiconductors (using gallium arsenide and arsine gas) and glass making. Inorganic arsenic and the methylated metabolite DMA can be found in rice and rice products [13,14]. The most common non-rice based dietary arsenic compounds are arsenobetaine [AB], arsenocholine [AC], trimethylarsine oxide [TMAO] and arsenosugars. These dietary species are found in marine algae, squid, crustaceans, bivalves and fish [15,16]. Humans are unable to further metabolise AB and AC after ingestion [17,18,19], as a result of four stable carbon bonds making them quasi-inert [19]. Subsequently, AB and AC are excreted in the urine unchanged and assumed to be completely non-toxic [17,18,19]. Arsenosugars, unlike AB and AC, are bio-accessible and can be transformed into other metabolites after human ingestion. These possible metabolites are DMA^5+^ (the major metabolite) and numerous DMA analogues: dimethylarsenoacetate [DMAA]; dimethylarsenoethanol [DMAE]; thio-dimethylarsenoacetate [thio-DMAA]; thio-dimethylarsenoethanol [thio-DMAE]; and thio-dimethylarsinate or dimethylthioarsinic acid [thio-DMA / DMTA]. These compounds are named dimethylarsinoyl compounds where the arsinoyl group represents either As=O or As=S and so are commonly termed oxo- and thioarsenicals [20]. Both monomethylated and di-thiolated forms such as dimethyldithioarsinic acid [dithio-DMA/DMDTA], monomethylthioarsonic acid [thio-MMA/MMTA] and monomethyldithioarsonic acid [dithio-MMA/MMDTA] have also been proposed [21,22,23,24,25].

The first study to report a thioarsenical metabolite in “natural” biological samples was from the urine of sheep that inhabit the beaches of North Ronaldsway (Orkney Island, Scotland). These sheep were unique in that they are chronically exposed to arsenosugars as a result of their diet of seaweed [26]. The study determined and confirmed the thioarsenical using anion exchange high performance liquid chromatography (HPLC) coupled to an inductively coupled plasma mass spectrometry (ICP-MS) and electrospray mass spectrometry (ES-MS) [26]. Studies published by Francesconi’s research group [15,27,28] have reported the almost complete biotransformation of an ingested synthetically prepared arsenosugar, resulting in the excretion of a mix of twelve thio- and oxoarsenicals in urine. The investigation of these twelve arsenicals was performed using HPLC-ICP-MS and HPLC-ES-MS, with anion, cation or reverse phase chromatography to ensure full separation of all the arsenicals.

In later studies, thio arsenicals were reported in human urine samples from population studies [29,30,31]. These groups recognized the importance of accurate identification and employed ES-MS in addition to HPLC-ICP-MS. It is already known that DMA^5+^ is the major human urinary metabolite following both inorganic arsenic exposure and dietary arsenosugar exposure [32]; the literature implies perhaps the same premise for thio-DMA and possibly other thio-arsenicals being metabolites following both inorganic exposure or dietary arsenosugar exposure. This will be of some concern as toxicological studies have shown thio-DMA to be genotoxic, cytotoxic and as toxic or more cytotoxic than arsenite in studies with rats [33,34], human bladder cells [23,35,36] and human lung cells [37,38,39], with one study describing thio-DMA as “one of the most toxicologically potent arsenic species, relevant to arsenic induced carcinogenicity” [23].

Since 2010, the Health and Safety Executive’s (HSE’s) Health and Safety Laboratory, based in Buxton, England, has been using a sensitive, robust and reliable speciation method for determining five species of arsenic in urine (arsenobetaine [AB], arsenite [As^3+^], arsenate [As^5+^], monomethylarsonic acid [MMA^5+^] and dimethylarsinic acid [DMA^5+^]) using a micro LC system coupled to an ICP-MS with a specialty anion exchange micro column. In this study, we investigate two different unexpected arsenic peaks observed in the analysis of urine samples from forensic cases, occupational exposure, hospital patients and as part of a background level study. The two unexpected peaks have been termed peak 1 and peak 2. Identification was facilitated using micro liquid chromatography coupled to ICP-MS (µLC-ICP-MS) and electrospray time of flight tandem mass spectrometry (ESI-Qq-TOF-MS/MS).

Previous unidentified DMA analogues which include; DMAA, DMAE, thio-DMAA, thio-DMAE and thio-DMA have been reported in urine of specific population studies exposed to inorganic arsenic contaminated drinking water [29,31], populations exposed to excessive amounts of arsenosugars [26,30,40] and in specific human dosing or ingestion studies [15,27,28,41,42]. However, this is the first study to report the observation of these similar or same arsenic compounds not from specific epidemiological populations, but routine biological monitoring urine samples. These samples are from a variety of industrial companies and geographical locations with no unifying commonalties as to the source of exposure to help aid either the identification of the arsenic species or with interpretation/feedback for customers. Based on an extensive review of the literature and on the availability of arsenic compounds, seven arsenicals (DMAA, DMAE, thio-DMAA, thio-DMAE, thio-DMA, TMAO and AC) were used to help determine the two unexpected arsenic peaks. These two arsenic peaks to date have not been observed at the same time in the same urine sample. Arsenic structures in addition to the structures of the seven arsenic compounds investigated in this study can be found in Figure S1 in the supplementary information.

## 2. Materials and Methods

### 2.1. Standards and Reagents

Arsenic speciation compounds sodium arsenite (NaAsO_2_), sodium arsenate (Na_2_HAsO_4_.7H_2_O) and sodium cacodylate (DMA; Na(CH_3_)2AsO_2_.3H_2_O) were all from Fisher Scientific (Loughborough, UK). Disodium methyl arsenate (99% MMA; CH_3_AsO(OH)_2_) was from ChemService (West Chester, PA, USA). Arsenobetaine (AB; C_5_H_11_AsO_2_) was from Sigma-Aldrich (Dorset, UK). Arsenocholine (AC; C_5_H_14_AsBrO) and trimethyl arsine oxide (TMAO; C_3_H_9_AsO) were both from Argus Chemicals (Vernio, Italy). Analytical quantities of the oxoarsenicals and thioarsenicals dimethylarsenoacetate (DMAA), dimethylarsenoethanol (DMAE), thio-dimethylarsinate (thio-DMA; (CH_3_)2As(S)OH), thio-dimethylarsenoacetate (thio-DMAA) and thio-dimethylarsenoethanol (thio-DMAE) were all synthesized by Kevin Francesconi’s group at Karl-Franzens University Graz. Ammonium carbonate and hydrogen peroxide were from VWR (Leicestershire, UK). All water used was ultrapure deionized water (18.2 MΩhm cm) from a Millipore system (Merck Millipore, Billerica, MA, USA).

Single standard stock solutions of 1000 mg·L^−1^ of AB, As^3+^, As^5+^, DMA, MMA, AC and TMAO were prepared in 1% (*v/v*) nitric acid, and stored at 4 °C. The analytical grade quantities of DMAA, DMAE, thio-DMAA, thio-DMAE and thio-DMA were reconstituted with 1 mL of water. Individual aliquots of 200 µL were stored at −80 °C until analysis.

### 2.2. Instrumentation

#### 2.2.1. Species Identification by µLC-ICP-MS

Separation was performed using a hyphenated µLC system with an ICP-MS (XSERIES 2, Thermo Fisher Scientific, Hemel Hempstead, UK). The separation of arsenic species was achieved using a 5 cm anion exchange guard column, (Dionex IONPAC AG7 4 mm × 50 mm i.d., 10 µm, Thermo Fisher Scientific). The micro-flow delivery of sample and mobile phase was accomplished using an ESI OneFAST system (Elemental Scientific, Warrington, UK), which consists of a six port switching valve and 1 mL sample loop. The ICP-MS was operated in collision cell (CCT) mode using 7% hydrogen in helium (approximately 3.5 mL/min), RF power 1400 W, using a Ni Xt skimmer and a Pt sample cones.

The delivery of mobile phases and sample onto the column was controlled by the ICP-MS peristaltic pump at a constant 0.2 mL/min. The µLC-ICP-MS method used comprises of a step gradient using two mobile phases of 2 mM ammonium carbonate (mobile phase A) and 70 mM ammonium carbonate (mobile phase B). Total acquisition time is 360 s with a sample injection time of 10 s. Mobile phase A is the carrier solution, 120 s after the injection of the sample, mobile phase B is injected for 120 s.

#### 2.2.2. Species Identification by ESI-QqTOF-MS/MS

Molecular identification was performed using an electrospray ionization quadrupole-time of flight (ESI-QqTOF) mass spectrometer (QStar XL MS-MS, Applied Biosystems, Concord, ON, Canada) with a turbo ion-spray source. The MS/MS experiments were carried out in positive ion mode with a collision energy of 20 eV using argon as the collision gas. Samples were injected separately into the ESI-QqTOF-MS using syringe infusions.

### 2.3. Sample Collection

All participating individuals were provided with informed consent, in accordance with HS(G) 167 [43]. All urine samples analyzed for arsenic speciation were received at the Health and Safety Laboratory as part of the analytical service for biological monitoring to assess arsenic exposure. From 2012 to 2016, an unexpected peak was observed in 4% of urine samples analyzed. The urine samples arrive with little contextual data apart from name, date of birth and details of the sender. Urine samples containing an unidentified arsenic peak were from UK semi-conductor workers, South African miners, forensic incidents, hospital patients and a participant of an arsenic-unexposed background level study [44]. It is important to note that urine samples from South African Miners and hospital patients were only received at the Health and Safety Laboratory for arsenic speciation analysis if total arsenic concentrations exceeded the US biological exposure index (BEI) [45] of 35 µg·L^−1^ (approximately 40 µmol/mol creatinine) at other laboratories. Upon receipt at the laboratory all urine samples for arsenic speciation are stored frozen at approximately −20 °C until analysis. For this study, samples that contained an unexpected peak had 1 mL aliquots taken and stored at −80 °C until identification analysis. Urine samples are not filtered prior to analysis and sample preparation involves only a 15-fold dilution with mobile phase A.

## 3. Results

### 3.1. Identification of Unexpected Arsenic Peaks by µLC-ICP-MS

**Figure 1:**
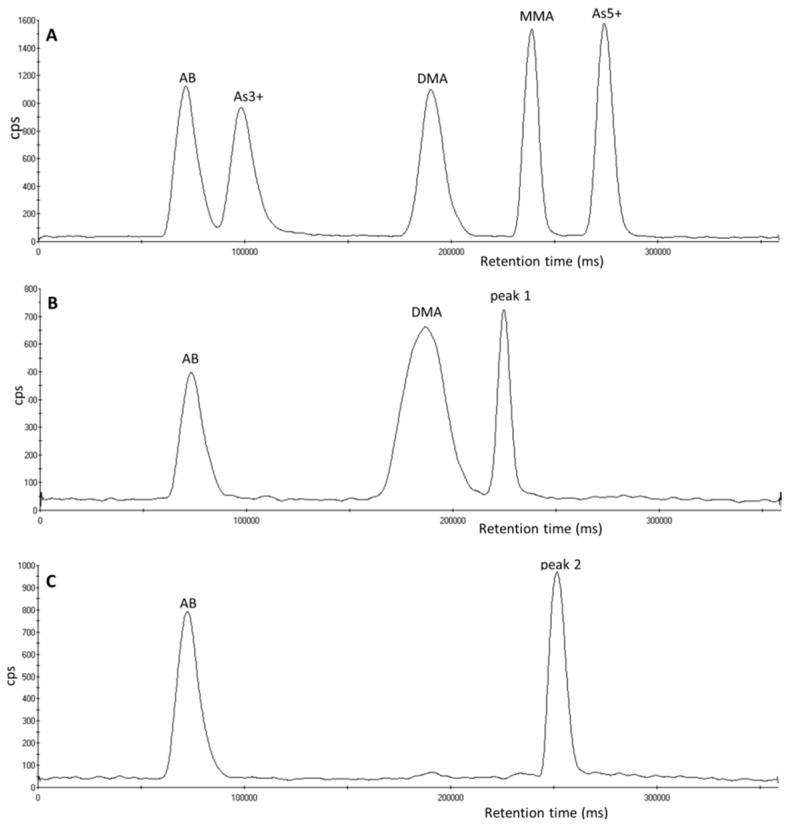
Chromatograms of arsenic species analyzed using an ESI OneFAST system coupled to a Dionex AG7 anion exchange column and ICP-MS using mobile phases 2 mM and 70 mM ammonium carbonate solution (**A**) A 5 µg L^−1^ standard of the five arsenic species. (**B**) A urine sample containing peak 1. (**C**) A urine sample containing peak 2.

During analysis of urinary arsenic speciation, both peak 1 and peak 2 eluted at different retention times to the five arsenic species routinely determined at the Health and Safety Laboratory (AB, As^3+^, As^5+^, DMA and MMA) (Figure 1A). Figure 1 shows that peak 1 (Figure 1B) is eluted between DMA and MMA at a retention time of 216 s, peak 2 (Figure 1C) is eluted between MMA and As^5+^ at a retention time of 240 s.

The analytical quantities of DMAA, DMAE, thio-DMAA, thio-DMAE, thio-DMA at approximate concentrations of 20–100 µg·L^−1^, and 5 µg·L^−1^ standards of AC and TMAO were injected onto the Dionex AG7 column. It was possible to observe each of the seven additional arsenic compounds with the existing µLC-ICP-MS method, although not all species would be fully separated if injected together. Figure S2 in the supplementary information shows the elution retention times of each of these six oxo- and thioarsenicals and AC when compared to the original five arsenic species.

### 3.2. Investigation of Peak 1 by µLC-ICP-MS

The urine samples analyzed where the arsenic peak, termed peak 1 was observed came from four different groups of individuals. The first group consisted of urine samples from two separate forensic cases, one of which was from a fatal arsenic poisoning where the approximate total arsenic concentration was 4800 µg·L^−1^ (–11,500 µmol mol creatinine) and contained the highest concentration of peak 1 in this study (approximate concentration 2800 µg·L^−1^). The second group consisted of occupational samples from South African miners, where 33% of urine samples analyzed contained peak 1. The third group were from hospital patients within the UK, where the presence of peak 1 accounts for 21% of all hospital patient samples analyzed, and the fourth group was a volunteer who participated in a study to determine unexposed background reference values for five arsenic species in a UK population. The approximate concentration of peak 1 in other urine samples presented here were all less than 50 µg·L^−1^.

**Figure 2:**
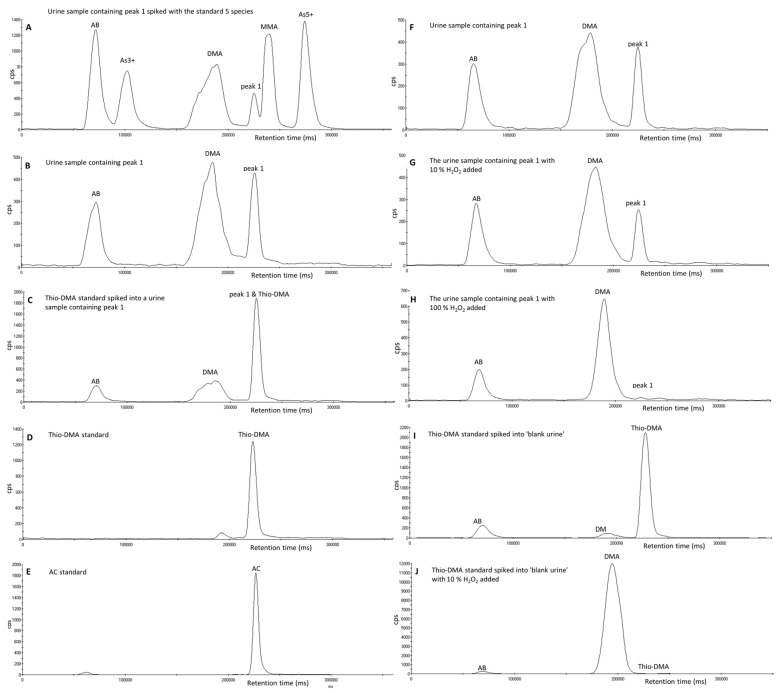
Chromatograms of arsenic species analyzed using an ESI OneFAST system coupled to a Dionex AG7 anion exchange column and ICP-MS using mobile phases 2 mM and 70 mM ammonium carbonate solution. (**A**) A urine sample containing peak 1 spiked with a 20 µg·L^−1^ solution of the standard five species. (**B**) An unspiked urine sample containing peak 1. (**C**) The urine sample containing peak 1 spiked with an approximate concentration of 40 µg·L^−1^ thio-DMA standard. (**D**) A thio-DMA standard at an approximate concentration of 40 µg·L^−1^ and (**E**) A 5 µg·L^−1^ AC standard. (**F**) The urine sample containing peak 1. (**G**) The urine sample with 10% *v/v* H2O2 added (**H**) The urine sample with 100% H_2_O_2_ added (**I**) Thio-DMA standard spiked into ‘blank urine’ not containing peak 1 (**J**) The spiked urine sample from chromatogram D with 10% *v/v* H_2_O_2_ added.

The urine samples that contained peak 1 varied in pH from pH 4–pH 7.2. These differences in pH of the urine samples did not affect the retention time of peak 1. The chromatogram of a urine sample containing peak 1 is shown in Figure 2B. When a urine sample containing peak 1 was spiked with a 20 µg·L^−1^ solution containing the standard five species, peak 1 eluted separately from the other five arsenic species, however as shown in Figure 2A, peak 1 elutes on the shoulder of the MMA peak. Of the six oxo- and thioarsenicals (DMAA, DMAE, thio-DMA, thio-DMAA and thio-DMAE) and AC injected onto the column, thio-DMA and AC were the only species that had a similar retention time to peak 1 (see Figure 2B,D,E). When the urine sample containing peak 1 was spiked with thio-DMA at an approximate concentration of 90 µg·L^−1^, the peak at the retention time of peak 1 increases (see Figure 2C).

When 10% *v/v* of H_2_O_2_ was added to a urine sample containing peak 1, the height of peak 1 reduced while the DMA peak height increased (see Figure 2F,G). When this was repeated with 100% of H_2_O_2_, peak 1 completely disappeared and the DMA peak increased accordingly (see chromatogram H of Figure 2). When thio-DMA was treated with 10% *v/v* H_2_O_2,_ the thio-DMA peak was completely converted to a peak at the same retention time as DMA (see Figure 2I,J). When AC was treated with 100% of H_2_O_2_ the AC peak did not change retention time or reduce in peak height nor was there an effect on any of the other peaks. Therefore, this is evidence that peak 1 is not AC.

**Figure 3:**
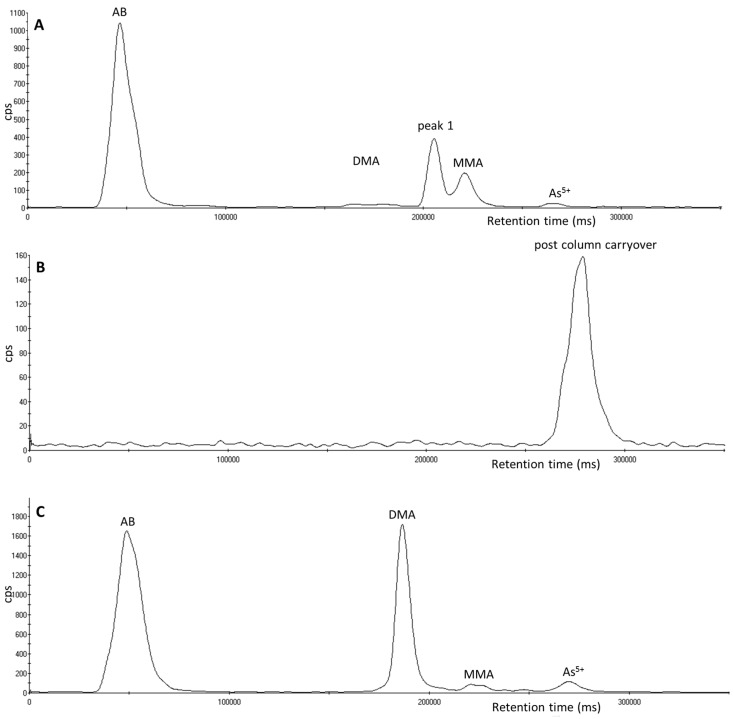
Chromatograms of arsenic species using an ESI OneFAST system coupled to a Dionex AG7 anion exchange column and ICP-MS using mobile phases 2 mM and 70 mM ammonium carbonate solution. (**A**) A urine sample from a hospital patient containing peak 1. (**B**) The post column carryover peak of an unknown into the blank sample immediately after the urine sample containing peak 1. (**C**) Chromatogram of the 5-day-old urine sample with 100% H_2_O_2_ added.

Interestingly, one particular urine sample containing peak 1 received from a hospital patient also showed the presence of an arsenic compound that eluted post column and was observed as a carryover peak following the injection of mobile phase B in the following sample. A repeat injection of the urine sample followed by the injection of a mobile phase A blank sample showed the presence of the same peak as shown in Figure 3. Unfortunately, further investigation of this peak was prohibited due to conversion of this peak to peak 1 and DMA during weekend storage in the fridge at 4 °C. The addition of 100% H_2_O_2_ converted the very small remainder of this parent compound and all of peak 1 to DMA.

### 3.3. Investigation of Peak 1 by ESI-QqTOF-MS/MS

AC was not thought to be the identity of peak 1 because the arsenic compound termed peak 1, was affected by the addition of H_2_O_2_ and AC was not. Early investigations using tandem mass spectrometry (not cited in the methodology) showed that when monitoring the protonated molecule [M + H]^+^ *m/z* 165 of AC, no signal was observed at the retention time of an AC standard, when the urine sample containing approximately 2800 µg·L^−1^ of peak 1 was injected into the MS/MS.

**Figure 4:**
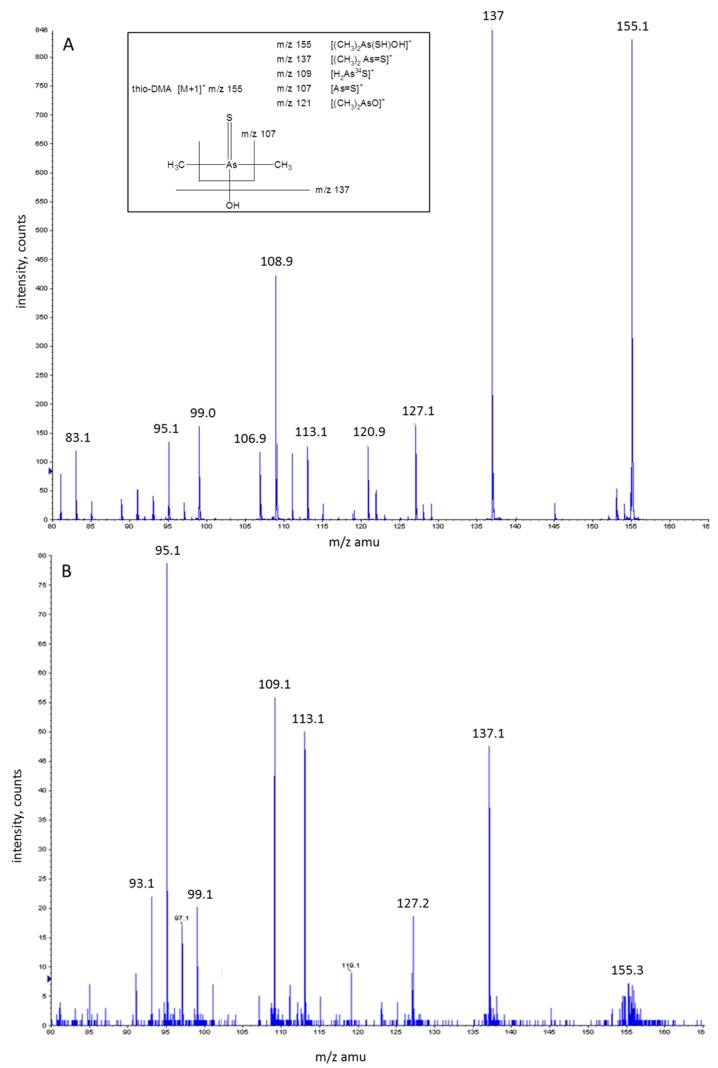
The chemical structure of thio-DMA, with suggested fragment ions when measured in positive ion mode by ESI-QqTOF-MS/MS within the two ESI-QqTOF-MS/MS spectra from product ion scans of *m/z* 155, when infusing (**A**) Thio-DMA standard at an approximate concentration of 1600 µg·L^−1^. (**B**) Fraction of a urine sample containing peak 1 with blank urine correction from a urine sample not containing peak 1.

While the µLC-ICP-MS investigations of peak 1 had indicated that this peak was thio-DMA, further confirmation was sought using mass spectrometry. As shown in Figure 4, the [M + H]^+^ ion of thio-DMA is *m/z* 155, with expected product ions of *m/z* 137, 121, 109, 107 and 75.

Since it was not possible in our laboratory to simultaneously analyze the arsenic species by coupling µLC-ICP-MS with ESI-QqTOF-MS/MS, samples had to be analyzed separately using ESI-QqTOF-MS/MS. The samples were injected into the ESI-QqTOF-MS/MS using syringe infusion. To undertake this, an aqueous standard solution of thio-DMA of approximate concentration of 1600 µg·L^−1^ was injected into the ESI-QqTOF-MS/MS (see Figure 4A). In a product ion scan of *m/z* 155, signals at *m/z* 155, 137 and 109 were observed, and additionally a small product ion at *m/z* 107. Other minor peaks that were observed in the spectra of the thio-DMA standard were *m/z* 127, 113, 99 and 95. In addition, a urine sample containing peak 1 has been fraction collected using the LC system on the ICP-MS and the fraction containing peak 1 was injected into the ESI-QqTOF-MS/MS (see Figure 4B). Again, the product ion mass spectrum of *m/z* 155, gave peaks at *m/z* 155, 137, 109 corresponding to the product ion spectrum of the thio-DMA standard. The dominant peak was *m/z* 95 with intermediate peaks at *m/z* 137, 113 and 109 and with smaller peaks at *m/z* 155 and 127, all of which concurred with the spectra of the thio-DMA standard.

### 3.4. Investigation of Peak 2 by µLC-ICP-MS

The urine samples containing the unexpected arsenic peak termed peak 2 came from two different groups of individuals. The first group were from different hospital patients, in separate hospitals within the UK. The symptoms of both individuals varied but both were surmised to have ingested an excessive amount of herbal remedies purchased online. The two individuals had consumed a different herbal remedy, one was kelp powder, administered orally by adding to food, the ingredient of the second herbal remedy was unknown, but was a black colored tablet administered orally. In total peak 2 has been observed in 9% of all hospital samples analyzed. The second group consisted of occupational urine samples from UK semi-conductor workers, peak 2 has been observed in 1% of all urine samples from semi-conductor workers.

**Figure 5:**
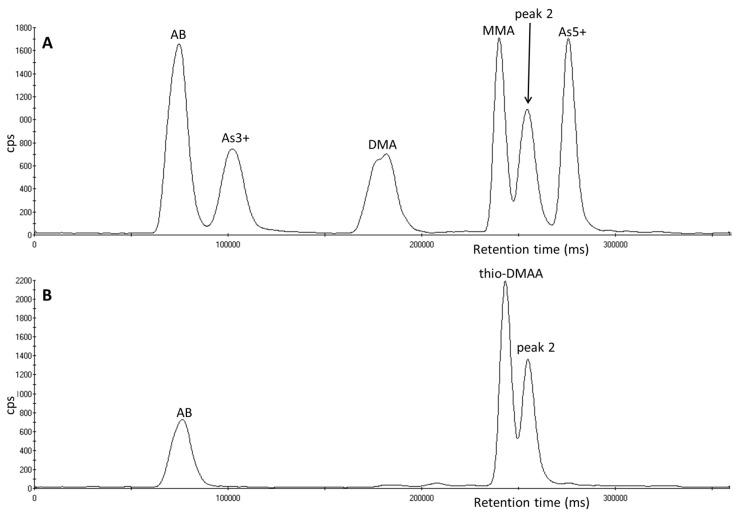
Chromatograms of arsenic species analyzed using an ESI OneFAST system coupled to a Dionex AG7 anion exchange column and ICP-MS using mobile phases 2 mM and 70 mM ammonium carbonate solution. (**A**) Chromatogram of a urine sample containing peak 2 spiked with a 20 µg·L^−1^ standard mix of the five species of arsenic. (**B**) Chromatogram of the urine sample spiked with thio-DMAA.

The urine samples that contained peak 2 varied in pH, from pH 5.5–pH 7.8. These differences in pH of the urine samples did not affect the retention time of peak 2. When a urine sample containing peak 2 was spiked with a 20 µg·L^−1^ five species standard mix of arsenic species, peak 2 eluted separately from the other five arsenic species (see Figure 5A). Of the six oxo- and thioarsenicals (DMAA, DMAE, thio-DMA, thio-DMAA and thio-DMAE, TMAO) and AC injected onto the column, none matched the retention time of peak 2; however thio-DMAA is the closest. When a urine sample containing peak 2 was spiked with a standard of thio-DMAA, although not fully separated, two unresolved peaks were observed (see Figure 5B).

When thio-DMAA was treated with 50% *v/v* H_2_O_2_, the thio-DMAA peak was mostly converted to a peak at the same retention time as DMAA, leaving a much reduced sized peak of thio-DMAA. However when a urine sample containing peak 2 is treated with 100% H_2_O_2_, the peak does not reduce or change in retention time and there is not a peak at the location assigned to DMAA. Chromatograms are shown in Figure S3 in the supplementary information.

### 3.5. Investigation of Peak 2 by ESI-QqTOF-MS/MS

Considerable efforts were made to analyze the urine samples with peak 2 by ESI-QqTOF-MS/MS. Two different tandem mass spectrometry instruments were used, different solvents were investigated and the fraction-collected peaks were freeze dried and reconstituted in an attempt to concentrate the samples. However, due to the inherent problems surrounding the difficulty in ionizing oxo- and thioarsenicals by MS-MS, there was insufficient sensitivity using the ESI-QqTOF-MS/MS to identify the protonated [M + H]^+^ ion of thio-DMAA at *m/z* 197, or its expected product ions of *m/z* 165, 151, 137, 107 and 75. Using negative ion mode was also unsuccessful. No definitive signal was observed across the mass range. The approximate concentration of the arsenic compound of peak 2 in all the urine samples used in this study was considered too low (estimated concentrations < 50 µg·L^−1^) to detect any [M + H]^+^ or product ions which corresponded to DMAA, DMAE, thio-DMAA, thio-DMAE or thio-DMA. To aid identification of peak 2, no other analytical techniques were investigated as it was deemed that the ESI-QqTOF-MS/MS would have the lowest LOD to facilitate the identification of arsenic compounds at such low concentrations.

To date we have been unable to identify peak 2, however this arsenic compound seems stable in urine, and to date has not converted to another peak when stored for 12 months frozen at either −80 °C or −20 °C.

## 4. Discussion

The investigation, by both µLC-ICP-MS and ESI-QqTOF-MS/MS, has determined that peak 1 is thio-DMA. Unfortunately, peak 2 has not been identified, although it has been possible to eliminate seven arsenic species in the process. This is not the only study to report unsuccessful identification of arsenic peaks in similar investigations [15,27,28,41,43,46]. While a human dosing study reported the almost complete biotransformation of an ingested synthetically prepared arsenosugar (2,3-dihydroxypropyl 5-deoxy-5-dimethylarsinoyl-β-D-riboside), which resulted in the excretion of twelve arsenicals in urine [15,27,28], initially only three of the twelve could be identified [15], which were DMA^5+^, TMAO and DMAE. Repeat studies further identified DMAA, thio-DMAA, thio-DMAE, the arsenosugar and thio-arsenosugar and trace amounts of thio-DMA and TMAS, with two species remained unidentified [27,28]. All information in this challenging analytical field is valuable and contributes to the scientific knowledge and future studies of a similar nature.

The urine samples which contained thio-DMA (peak 1) were obtained from four sources consisting of two separate forensic cases, occupational exposure samples, a hospital patient and an individual participating in a background levels study. Only the urine samples from the two forensic cases had clear evidence of inorganic arsenic exposure (inorganic arsenic and elevated DMA^5+^ and MMA^5+^ were observed during speciation analysis). The urine sample of the additional hospital patient saw the elution of a further arsenic compound eluting post column in the following sample (Figure 3). This compound is potentially a “parent” arsenic compound and it was found to be quite unstable, as it had completely converted to thio-DMA after refrigeration at 4 °C and subsequently DMA^5+^ after the addition of hydrogen peroxide. The presence of this parent compound has not been observed previously or since, nor has it been observed in urine in the current literature to provide an identification suggestion. It is common knowledge in the literature [23,25,26,30,38,47] that treatment of DMA^5+^ with hydrogen sulphide will produce thio-DMA. Furthermore, it is known that continued treatment of thio-DMA with hydrogen sulphide will produce dithio-DMA [47,48] and the subsequent addition of hydrogen peroxide can convert dithio-DMA back to thio-DMA [49]. The study by Conklin [49] found the conversion between DMA, thio-DMA and dithio-DMA in aqueous standards was pH specific, with DMA only converting to its thio-analogues when the pH range was between pH 5–7. Unfortunately, in our study, due to the rapid degradation of this observed parent compound in the urine sample and the lack of a dithio-DMA standard to match retention time, the authors were unable to make any initial analytical confirmation of the identity of the compound and can therefore only postulate the parent compound’s identity as dithio-DMA.

It is possible that the source of peak 1 in the urine samples from the South African miners is either from dietary or occupational sources. However, in these samples inorganic arsenic was not observed, with the dominant species being DMA^5+^ and MMA^5+^ with smaller concentrations of AB. These mixed findings of both inorganic exposure and dietary arsenosugar exposure are similar to those found in the literature. A study looking at women in Bangladesh exposed to inorganic arsenic in their drinking water found that 44% of urine samples from 75 women contained thio-DMA [31]. A similar study looking at a population exposed to inorganic arsenic through drinking water in West Bengal found thio-DMA in both urine and nails [29]. A noteworthy study by Raml [30] in 2006 determined thio-DMA, thio-DMAA and thio-DMAE in a human urine reference material, NIES 18. The thioarsenicals were reported to represent approximately 10% of the total urinary arsenic. Interestingly, the human urine used to produce this reference material is not spiked with arsenic species but is collected from Japanese men who were not occupationally exposed to arsenic, but whose diet is rich in seafood, algae and mollusks [30]. Studies have also shown that inorganic arsenic can be converted to thioarsenicals in ground water [48] and algae has also shown to be able to convert inorganic arsenic into both methylated oxo- and thioarsenicals [48], with arsenosugars being the major arsenic compound found in marine algae [50]. This goes some way to explain why thio-DMA is found in human urine from a variety of possible sources of exposure.

The ESI-QqTOF-MS/MS analysis in this study of thio-DMA is in agreement with the LC-ESI-MS/MS analysis of a thio-DMA standard by Fricke [47], who also found product ions at *m/z* 137, 121 and 109. However, the ESI-QqTOF-MS/MS analysis in this study also produced product ions corresponding to *m/z* 127 and 113. The peak at *m/z* 127 is in agreement with Hansen [51] however the actual molecular structure for this signal and *m/z* 113 is unknown.

A study that found an unknown arsenic peak during the speciation of a rice extract (from rice cooked in arsenic contaminated water), also determined their unknown peak to be thio-DMA [52]. They employed the same analytical techniques used in this study to determine its identity; thio-DMA spikes, conversion using H_2_O_2_ and IC-ESI-MS analysis. However, they used negative ion mode MS, meaning peaks were identified at *m/z* 153, 138, 123 and 105.

The urine samples which contained peak 2 came from two sources consisting of two separate hospital patients and UK semi-conductor workers. The only other arsenic species present in both the urine samples of the two separate hospital patients apart from peak 2 was arsenobetaine. This would suggest that the presence of peak 2 was not from inorganic arsenic exposure but most likely from dietary exposure. Furthermore, while the source of exposure for the semi-conductor workers potentially could be occupational inorganic exposure, the speciation analysis of their urine samples indicated otherwise.

Several studies have reported differences in the excretion of oxo- and thioarsenical metabolites after the ingestion of arsenosugars [28,40,53]. Suggested possible reasons for this human metabolic variation might be differences in biotransformation enzymes found in the liver, people’s differing ability to transform or degrade arsenosugars, differences in gut flora or the ability to uptake arsenosugars [28]. This individual metabolic variation/uniqueness may suggest why these oxo- and thioarsenicals are not seen as frequently as other dietary compounds such as AB during our general arsenic speciation analysis at the Health and Safety Laboratory.

The results outlined in Figure 2F–J and Figure S3 show that when thioarsenicals, thio-DMA and thio-DMAA, are oxidized with H_2_O_2_ they begin to convert to their oxo analogues; DMA^5+^ and DMAA respectively. However as shown in Figure S2C and Figure S2F, thio-DMAE and DMAE have identical retention times using the chromatographic conditions used in this study, therefore it is difficult to say if the same oxidation process also applies to thio-DMAE. The fact that peak 1 (thio-DMA) follows this same oxidative process and peak 2 does not, may suggest that peak 2 is not a thioarsenical; this hypothesis would agree with the literature where it is stated that the oxidizing agent hydrogen peroxide converts thioarsenicals to their oxoarsenical analogues [27,48]. Therefore, peak 2 could be one of the oxoarsenicals or an arsenosugar not analyzed in this study. The study by Raml [27] reported traces of an arsenosugar in urine but it was not sufficiently separated from the other arsenicals when using an anion exchange column. In addition a study in 2006 by Schmiesser [42] found arsenolipid metabolites in human urine after the ingestion of cod liver, known as arsenic fatty acids such as dimethylarsenopropanoic acid (DMAP), thio-dimethylarsenopropanoic acid (thio-DMAP), dimethylarsenobutanoic acid (DMAB) and thio-dimethylarsenobutanoic acid (thio-DMAB), with all but DMAP were determined using anion exchange chromatography.

Several research groups have recently attempted to better explain arsenic metabolic pathways using the liver of rats [54] and rat or mouse cecum [48,55]. Wang [48] suggests that pre-absorptive methylation of thioarsenicals is facilitated by gastrointestinal microbiota before transport across the gastrointestinal barrier, and post-absorptive formation of thio-arsenicals occurs when oxoarsenicals are converted due to the presence of H_2_S formed in tissues. They also postulated that during in-vitro reactions both thio-DMA and dithio-DMA occur from DMA^3+^, while in-vitro human red blood cells take up DMA^3+^ and quickly convert it to thio-DMA. This research does advance the understanding of these pathways but it is worth noting that Wang [48], Suzuki [54] and Kubachka [55] have all proposed different possible pathways for the in vitro methylation, reduction and thiolation of inorganic arsenic as well as to mono-, di- and trimethylated species with a range of oxo- and thioarsenical products.

## 5. Conclusions

In conclusion, the investigation, by both µLC-ICP-MS and ESI-QqTOF-MS/MS, has determined that peak 1 is thio-DMA. Peak 2 has not been identified to date. However, this study has established that peak 2 is not thio-DMA, DMAA, thio-DMAA, DMAE, thio-DMAE, AC or TMAO. The fact that peak 2 occurs with AB in the samples could suggest that its presence is a result of the consumption of seafood. In addition this study has shown that these two compounds occur in urine samples collected from normal and abnormal sources, they have not been observed in the same urine sample and that there may be a suggestion that thio-DMA is a breakdown product of dithio-DMA. The identification of these new arsenicals is extremely challenging for a combination of factors; there is a lack of available compounds; the sensitivity of organic mass spectrometric techniques used for the molecular identification of arsenic compounds and the overall knowledge of arsenic processes. Nevertheless, it is hoped that this study will inform and contribute to the continued research in oxo- and thioarsenicals in human urine samples.

## Supplementary Materials

The following are available online at www.mdpi.com/2305-6304/5/2/12/s1, Figure S1: Structures of inorganic arsenic, methylated metabolites, oxo- and thio- dimethylated analogues and dietary arsenic compounds. The structures in blue indicate the seven arsenicals investigated in this study, Figure S2: Chromatograms of seven additional arsenic species and their retention times analysed using an ESI OneFAST system coupled to a Dionex AG7 anion exchange column and ICP-MS using mobile phases 2 mM and 70 mM ammonium carbonate solution (A) the standard five arsenic species (B) DMAA, (C) DMAE, (**D**) thio-DMA, (E) thio-DMAA (F) thio-DMAE (G) AC and (H) TMAO, Figure S3: Chromatograms of arsenic species analysed using an ESI OneFAST system coupled to a Dionex AG7 anion exchange column and ICP-MS using mobile phases 2 mM and 70 mM ammonium carbonate solution. (A) A urine sample containing peak 2. (B) The urine sample with 100% *v/v* H_2_O_2_ added. (C) A thio-DMAA standard spiked into ‘blank urine’ (only containing AB arsenic species). (D) The spiked urine sample from chromatogram C with 50% *v/v* H_2_O_2_ added. (E) DMAA standard spiked into ‘blank urine’ (only containing AB arsenic species).
