# Supplementary material for: The Investigation of Unexpected Arsenic Compounds Observed in Routine Biological Monitoring Urinary Speciation Analysis
Source: Toxics. 2017 May 20;5(2):12. doi: 10.3390/toxics5020012 (PMC5606668; doi:10.3390/toxics5020012)
Supplement: Supplementary file 1 [file toxics-05-00012-s001.pdf]

Supplementary Materials: The Investigation of Unexpected Arsenic Compounds Observed in Routine Biological Monitoring Urinary Speciation Analysis

Elizabeth Leese, Malcolm Clench, Jackie Morton, Philip HE Gardiner and Vikki A Carolan

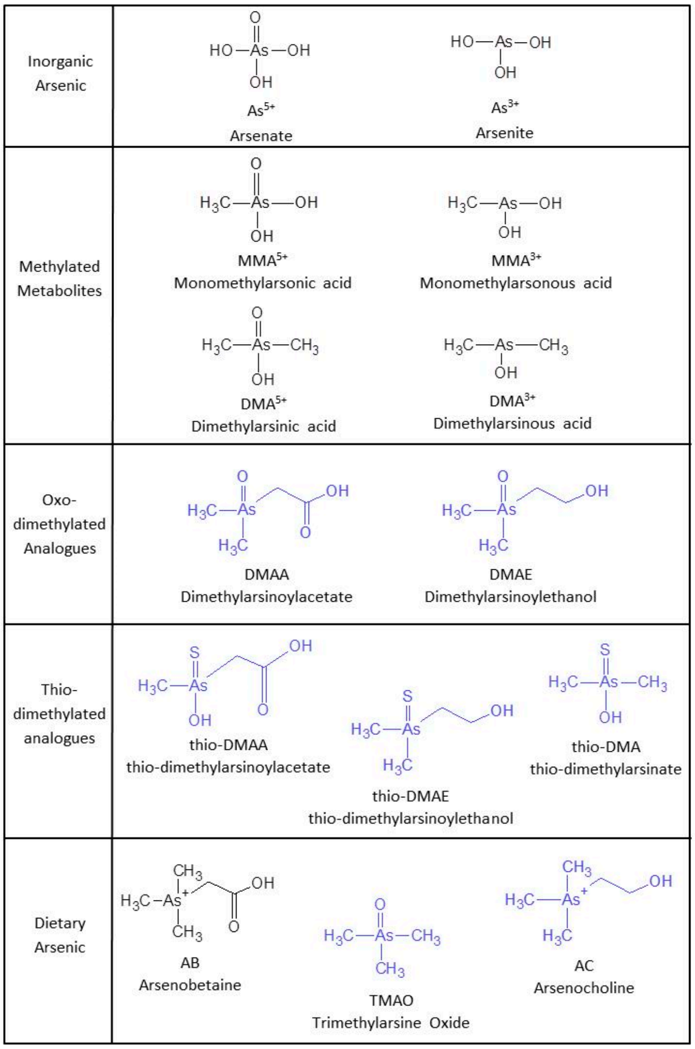

Figure S1. Structures of inorganic arsenic, methylated metabolites, oxo- and thio- dimethylated analogues and dietary arsenic compounds. The structures in blue indicate the seven arsenicals investigated in this study.

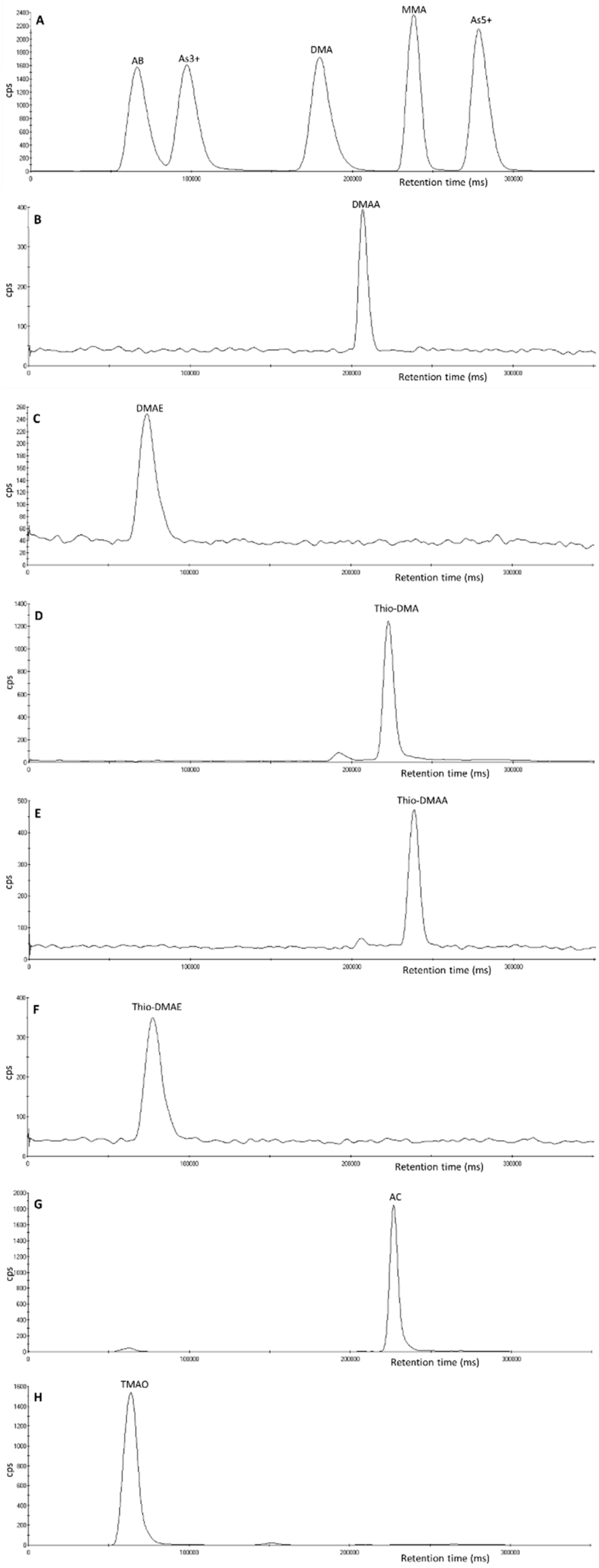

Figure S2. Chromatograms of seven additional arsenic species and their retention times analysed using an ESI OneFAST system coupled to a Dionex AG7 anion exchange column and ICP-MS using mobile phases 2 mM and 70 mM ammonium carbonate solution (**A**) the standard five arsenic species (**B**) DMAA, (**C**) DMAE, (**D**) thio-DMA, (**E**) thio-DMAA (**F**) thio-DMAE (**G**) AC and (**H**) TMAO.

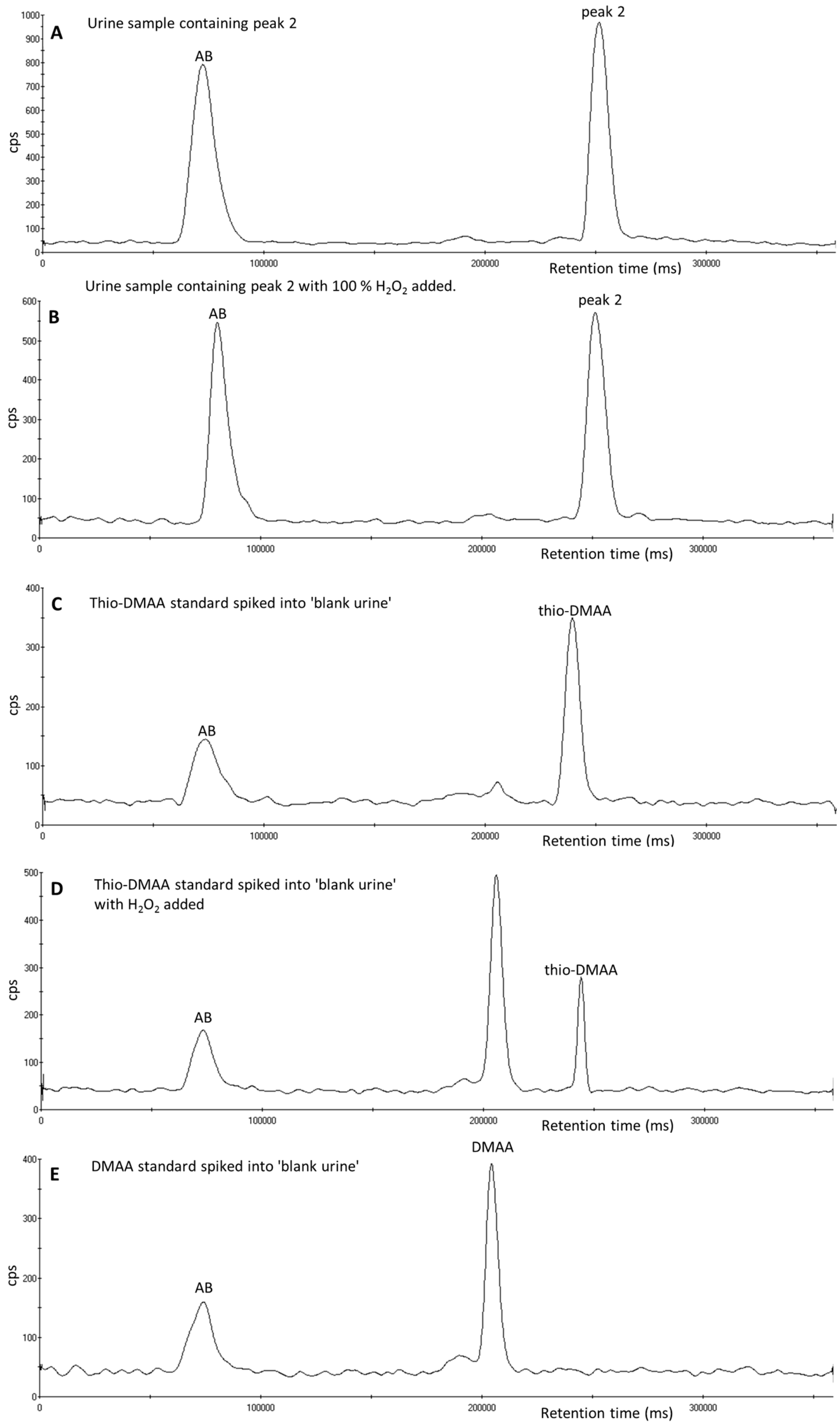

Figure S3. Chromatograms of arsenic species analysed using an ESI OneFAST system coupled to a Dionex AG7 anion exchange column and ICP-MS using mobile phases 2 mM and 70 mM ammonium carbonate solution. (**A**) A urine sample containing peak 2. (**B**) The urine sample with 100 % *v*/*v* H_2O_2 added. (**C**) A thio-DMAA standard spiked into 'blank urine' (only containing AB arsenic species). (**D**) The spiked urine sample from chromatogram C with 50 % *v*/*v* H_2O_2 added. (**E**) DMAA standard spiked into 'blank urine' (only containing AB arsenic species).
